# The Role of Mitochondrial Dynamics in Metabolic Dysfunction-Associated Steatotic Liver Disease and the Regulatory Mechanisms of Exercise Intervention: A Systematic Review of Preclinical Studies

**DOI:** 10.3390/metabo16010011

**Published:** 2025-12-23

**Authors:** Haonan Tian, Aozhe Wang, Haoran Wu, Lin Yan, Jun Wang

**Affiliations:** 1 School of Sport Science, Beijing Sport University, Beijing 100084, China; 2 Department of Exercise Physiology, Beijing Sport University, Beijing 100084, China; 3 School of Physical Education, Anhui Polytechnic University, Wuhu 241000, China

**Keywords:** exercise, mitochondrial dynamics, MASLD

## Abstract

**Background/Objectives**: Metabolic dysfunction-associated steatotic liver disease (MASLD) involves dysregulated mitochondrial dynamics. This review systematically integrates the specific mechanisms by which exercise modulates mitochondrial fusion, fission, and mass control in the liver within MASLD and metabolic dysfunction-associated steatohepatitis (MASH) models. **Methods**: A comprehensive search of PubMed and Web of Science identified 11 animal studies investigating exercise and mitochondrial dynamics markers. **Results**: MASLD generally exhibited a “pro-fission” phenotype. Exercise, particularly moderate-intensity continuous training (MICT) and high-intensity interval training (HIIT), reversed these alterations via “pro-fusion, anti-fission” effects and restored biogenesis and mitophagy. Crucially, effects appeared to be “modality-specific” and “intensity-dependent.” Current evidence suggests that reversing severe fission and restoring inner-membrane may require a specific “intensity threshold,” with voluntary wheel running showing limited efficacy in steatohepatitis. Notably, resistance exercise seemed to display a distinct profile, effectively curbing fission but diverging in fusion/biogenesis regulation. **Conclusions**: Synthesizing preclinical evidence, this review suggests that exercise ameliorates hepatic mitochondrial dysregulation in MASLD and appears to exhibit characteristics of “modality specificity” and “intensity dependence.” Specifically, an “intensity threshold” may be critical for profound structural remodeling, while resistance exercise exhibits a distinct regulatory profile. Future long-term clinical trials are warranted to validate these animal-derived findings and develop stage-specific “precision exercise prescriptions” for patients.

## 1. Introduction

Over the last thirty years, non-alcoholic fatty liver disease (NAFLD) has evolved into one of the most prevalent chronic liver diseases globally [1]. The global prevalence of NAFLD currently stands at 32.4%, with a marked gender disparity—higher in men (39.7%) than in women (25.6%)—and continues to rise annually [2]. Closely linked to obesity, type 2 diabetes, and other metabolic disturbances, NAFLD presents a critical public health challenge [3]. With growing insights into the pathological mechanisms of the disease, conventional diagnostic criteria have become insufficient for clinical requirements, hindering early diagnosis and effective intervention [4]. In 2020, an international panel proposed the term metabolic dysfunction-associated fatty liver disease (MAFLD) to better reflect the underlying pathophysiology [5]. More recently, to address potential stigmatization associated with the term “fatty” and to refine diagnostic criteria [6], a 2023 multi-society Delphi consensus led by the American Association for the Study of Liver Diseases (AASLD) and others established the nomenclature metabolic dysfunction-associated steatotic liver disease (MASLD) [7]. To ensure clarity and consistency, this review uses the term MASLD as the primary descriptor. Citations referencing earlier studies using the terms NAFLD or MAFLD have been interpreted within the context of the current MASLD definition.

MASLD encompasses a broad spectrum of liver pathologies, ranging from simple steatosis to metabolic dysfunction-associated steatohepatitis (MASH), fibrosis, cirrhosis, and ultimately, advanced hepatocellular carcinoma. While traditional investigations into MASLD pathogenesis have primarily centered on aberrant hepatic lipid metabolism, recent advancements in mitochondrial biology have unveiled the pivotal regulatory role of dysregulated mitochondrial dynamics in the disease’s onset and progression [8]. As the central hub of cellular energy metabolism, mitochondria maintain the plasticity of their network structure through a dynamic equilibrium of fusion and fission—a characteristic essential for adapting to metabolic stress and preserving cellular homeostasis. Disruption of this equilibrium can precipitate or exacerbate obesity-related metabolic disorders, thereby driving the development of MASLD. Consequently, targeting mitochondrial dynamics in hepatocytes, particularly the coordination between fusion and fission processes, has emerged as a critical frontier in deciphering MASLD mechanisms and exploring potential therapeutic strategies.

Given the limited efficacy and potential adverse effects associated with conventional pharmacotherapies, the exploration of effective non-pharmacological interventions is paramount. In this regard, exercise therapy stands out as a safe, sustainable, and cost-effective strategy that has been demonstrated to ameliorate mitochondrial morphological and functional impairments during the progression of MASLD, thereby mitigating disease severity [9,10]. Accordingly, this review summarizes the role of mitochondrial dynamics in MASLD and examines the regulatory mechanisms underlying exercise interventions, with the aim of offering novel insights and theoretical foundations for the design of personalized exercise prescriptions.

## 2. Materials and Methods

### 2.1. Search Strategy

A systematic literature search was conducted in two major biomedical databases, PubMed and Web of Science, to identify studies investigating the effects of exercise on mitochondrial dynamics in MASLD/MAFLD (last accessed on 15 November 2025). The search terms were constructed based on the PICOS principle, combining keywords related to the disease (e.g., “MASLD”, “MAFLD”, “NAFLD”, “Steatohepatitis”), mitochondrial dynamics (e.g., “Mitochondrial fission”, “Dynamin-related protein 1[Drp1]”, “Mitofusin 1/2[MFN1/2]”), and intervention (e.g., “Exercise”, “Aerobic”, “High-Intensity Interval Training [HIIT]”). Boolean operators (AND, OR, NOT) were utilized to refine the results. The specific search strings are detailed in Supplementary Table S1. The search was restricted to articles published in English, and reviews, systematic reviews, and meta-analyses were excluded during the initial database filtering. To ensure a comprehensive review, the reference lists of all included articles were manually screened to identify additional relevant studies.

**Figure 1:**
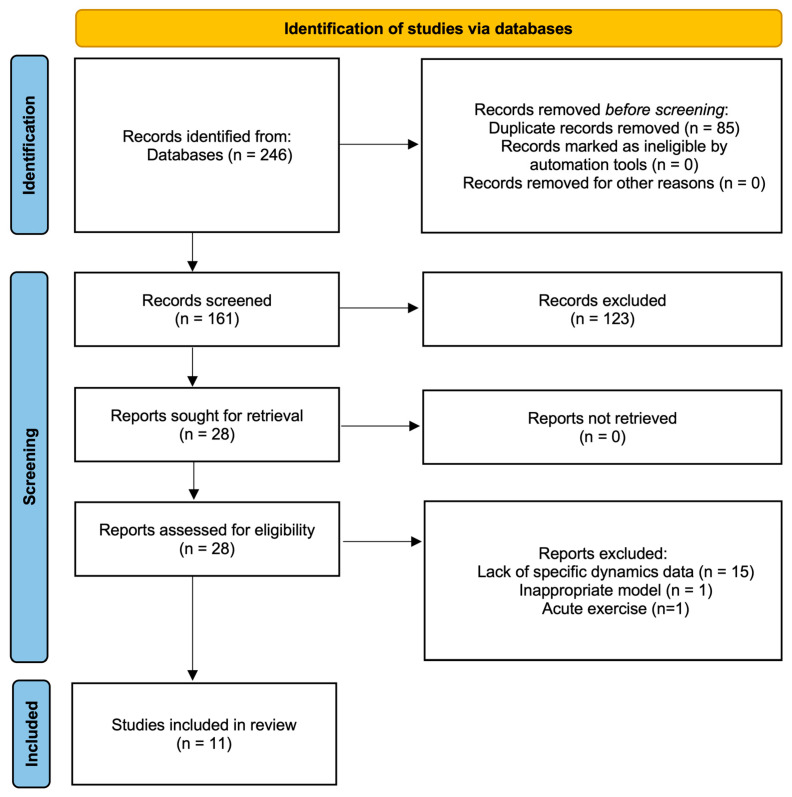
PRISMA flow diagram of study selection process.

This study was conducted in strict adherence to the Preferred Reporting Items for Systematic Reviews and Meta-Analyses (PRISMA) guideline [11,12]. The completed PRISMA checklist is available in Supplementary Table S2, and the study selection process is illustrated in the PRISMA flow diagram (Figure 1). The protocol for this review has been registered in the International Platform of Registered Systematic Review and Meta-analysis Protocols (INPLASY^®^) (Registration ID: INPLASY2025110097).

### 2.2. Inclusion and Exclusion Criteria

Studies were included if they met the following criteria: (1) Population: Animal models (rodents or zebrafish) with experimentally induced MASLD (formerly NAFLD/MAFLD). Given the recent nomenclature consensus, studies originally classified as NAFLD or MAFLD were included if they met the metabolic criteria consistent with the current MASLD definition. For uniformity, data from these studies are reported using MASLD (to denote simple steatosis) and MASH (to denote steatohepatitis) throughout this review. (2) Intervention: Chronic exercise training protocols (aerobic, resistance, or combined training) of any intensity, frequency, or duration. (3) Comparator: Sedentary control groups with the same disease induction. (4) Outcomes: Objective quantitative measurement of mitochondrial dynamics-related proteins or gene expression in liver tissue.

Studies were excluded if they (1) utilized acute exercise protocols (single bout); (2) used healthy animals without disease induction; (3) did not analyze liver tissue; or (4) were classified as gray literature, conference abstracts, editorials, or letters.

### 2.3. Study Selection and Data Extraction

The literature search was performed independently by two reviewers. All retrieved records were imported into Zotero (version 7.0.30; Corporation for Digital Scholarship, Vienna, VA, USA) reference management software, and duplicates were removed automatically. Following deduplication, the study selection was conducted independently by two reviewers. First, titles and abstracts were screened to exclude irrelevant studies. Subsequently, the full texts of the remaining articles were independently examined against the eligibility criteria by the same two reviewers. Disagreements at any stage were resolved by consensus or consultation with a third reviewer. Finally, data were independently extracted by two reviewers using a standardized form. Extracted information included: first author, publication year, animal species/strain, disease stage (MASLD/MASH), disease induction method, exercise protocol (type, intensity, duration, frequency), and alterations in mitochondrial dynamics markers, as well as other mitochondrial quality control (MQC) indicators.

### 2.4. Quality Assessment

The methodological quality of the included animal studies was assessed using the SYRCLE’s risk of bias tool for animal studies [13]. Two reviewers independently assessed the risk of bias across ten domains, including selection bias, performance bias, detection bias, attrition bias, reporting bias, and other sources of bias. Disagreements were resolved through discussion or consultation with a third reviewer.

### 2.5. Data Analysis

The preclinical studies incorporated in this review employed diverse animal models and disease induction protocols, thereby establishing distinct pathophysiological baselines. Concurrently, substantial heterogeneity exists across outcome measures, precluding quantitative pooling. For instance, detection methodologies varied between gene expression (qPCR) and protein abundance (Western blot). Given the potential discordance between mRNA and protein levels for identical markers, pooling data across these distinct biological endpoints could yield misleading conclusions. Moreover, mitochondrial dynamics indices are predominantly reported in a semi-quantitative manner lacking standardized reference systems, rendering the statistical calculation of merged effect sizes unreliable. Additionally, variability exists in the modalities and durations of the exercise protocols employed. In light of this significant heterogeneity in experimental designs and outcome metrics identified during data extraction, a quantitative meta-analysis was deemed inappropriate. Consequently, this study adopts a qualitative analysis approach to delineate the directionality and consistency of the observed effects [14,15].

## 3. Result

### 3.1. Identified Records

The initial database search yielded a total of 246 citations (88 from PubMed and 158 from Web of Science). After removing 85 duplicates, 161 records remained for title and abstract screening. Based on the preliminary screening, 123 articles were excluded (e.g., non-exercise interventions, no liver tissue analysis, reviews, or abstracts only). Consequently, 28 full-text articles were retrieved for detailed eligibility assessment. Of these, 17 were excluded for the following reasons: lack of mitochondrial dynamics markers (*n* = 13), purely subjective qualitative analysis via electron microscopy (*n* = 1), reporting Drp1phosphorylation without total protein (*n* = 1), use of healthy models without disease induction (*n* = 1), and acute exercise intervention (*n* = 1). Finally, 11 studies met all inclusion criteria and were included in this systematic review.

The included studies consisted of 5 rat models, 5 mouse models, and 1 zebrafish model. Regarding disease progression, most studies (*n* = 7) focused on the steatosis stage, three studies involved MASH, and one study covered both MASLD and MASH stages. In terms of exercise interventions, aerobic exercise was the predominant modality (*n* = 9), comprising varying intensities: three studies compared HIIT with Moderate-Intensity Continuous Training (MICT), one utilized HIIT exclusively, and five employed low-to-moderate intensity protocols (including voluntary wheel running [VWR]). Additionally, one study implemented a resistance training protocol, and one employed a combined aerobic and resistance training regimen.

### 3.2. Methodological Quality Assessment

**Figure 2:**
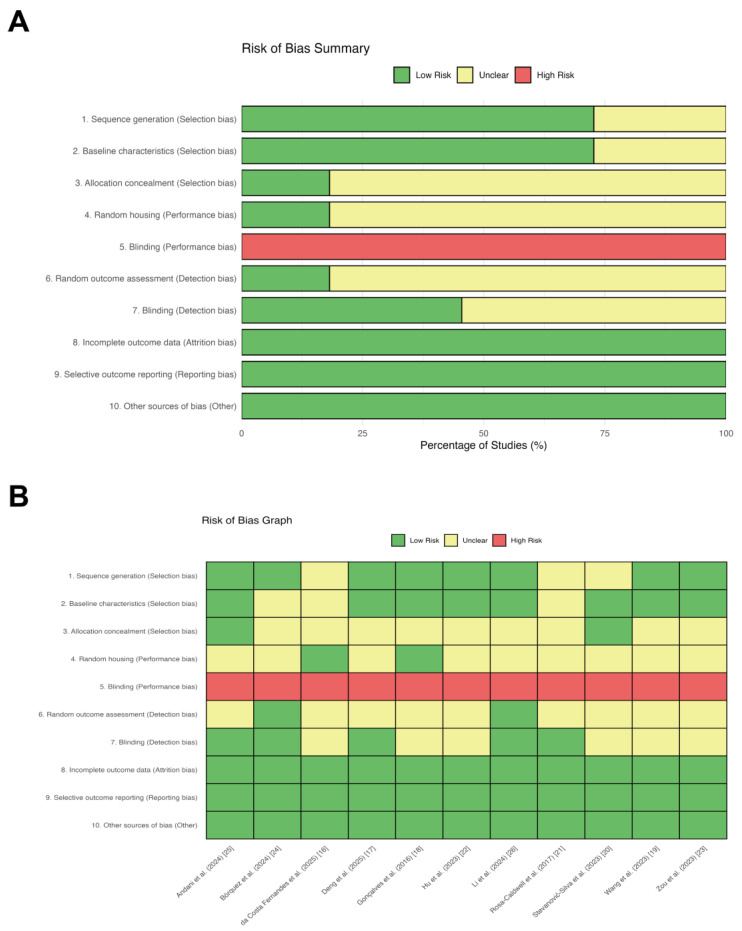
Detailed risk of bias assessment for each included study using SYRCLE’s risk of bias tool. (**A**) Aggregate risk of bias assessment across included animal studies. Review authors’ judgments about each risk of bias item presented as percentages across all included studies. The assessment was conducted using SYRCLE’s risk of bias tool. (**B**) Risk of bias assessment for individual studies. Detailed risk of bias assessment for each included study using the SYRCLE’s risk of bias tool. The colors represent the judgment of risk: green for low risk, yellow for unclear risk, and red for high risk.

The methodological quality of the included studies was rigorously evaluated using the SYRCLE risk of bias tool. The aggregate risk of bias is summarized in Figure 2A, and the assessment for individual studies is detailed in Figure 2B. Overall, the included studies demonstrated high reporting standards regarding attrition and reporting biases. All articles were rated as “low risk” for incomplete outcome data, selective outcome reporting, and other sources of bias. Regarding selection bias, the majority of studies exhibited a low risk for sequence generation and baseline characteristics, indicating effective randomization and comparable baseline traits among groups. However, details concerning allocation concealment were frequently omitted, resulting in an “unclear risk” rating for 82% of the studies. In terms of performance bias, all studies were classified as “high risk” for the blinding of caregivers and investigators. It is important to note that this is an intrinsic limitation of exercise physiology research, as the nature of the intervention (e.g., treadmill running) renders the blinding of personnel methodologically unfeasible. Consequently, this bias was deemed acceptable and did not warrant exclusion. Similarly, details on random housing conditions were largely unreported (82% unclear risk). Regarding detection bias, the random selection of animals for outcome assessment was rarely described (82% unclear risk). While only approximately 45% of the studies explicitly reported blinding during outcome assessment, this risk was partially mitigated by the widespread use of objective biochemical markers (e.g., Western blot, qPCR), which are less susceptible to subjective interpretation. In conclusion, the included studies demonstrated acceptable methodological quality for the purpose of this review.

### 3.3. Regulatory Effects of Exercise Intervention on Mitochondrial Dynamics

#### 3.3.1. Effects of Exercise on Mitochondrial Fusion Proteins

Mitochondrial fusion, primarily mediated by outer membrane proteins (MFN1, MFN2) and the inner membrane protein (OPA1), is essential for maintaining mitochondrial network connectivity and integrity. In pathological states of MASLD and MASH, fusion protein expression is generally suppressed, although subtle discrepancies exist across different models.

Multiple studies have demonstrated significant downregulation of MFN1 in MASLD and MASH (Table 1). Specifically, MFN1 expression was significantly reduced in the MASLD model [16,17], with the same phenomenon observed in the MASH model reported by Gonçalves et al. [18] and in the type 2 diabetes mellitus (T2DM) co-morbid MASH model studied by Wang et al. [19]. However, certain models, such as the gestational diabetes mellitus (GDM)-associated MASLD model described by Stevanović-Silva et al. [20] and the 8-week Western diet (WD)-induced model by Rosa-Caldwell et al. [21], did not exhibit significant alterations in MFN1. Regarding intervention efficacy, aerobic exercise demonstrated significant ameliorative effects. Deng et al. [17] reported that both HIIT and MICT reversed the downregulation of MFN1 protein in MASLD. Similarly, Gonçalves et al. [18] and Stevanović-Silva et al. [20] found that MICT significantly increased MFN1 protein levels. In contrast, VWR failed to induce significant changes in MFN1 protein across multiple studies [18,21]. Notably, da Costa Fernandes et al. [16] observed that RE resulted in a further suppression of Mfn1 mRNA expression.

MFN2, the most extensively studied fusion protein, demonstrated consistent downregulation across various MASLD induction protocols [17,20,21,22,23], including T2DM-comorbid MASH [19]. However, no significant changes in MFN2 were observed in some MASH models induced by high-fat diets or pharmacological agents [18,24,25]. Interestingly, da Costa Fernandes et al. [16] reported a significant increase in Mfn2 mRNA in a 14-week high fat diet (HFD)-induced MASLD model in Swiss mice. MICT has been confirmed to significantly upregulate MFN2 protein expression across multiple models [17,18,20,23,24], and combined training (RE + MICT) also showed efficacy in increasing MFN2 protein [22]. While Deng et al. [17] and Wang et al. [19] found that HIIT significantly increased MFN2 expression, Andani et al. [25] reported no significant changes following either MICT or HIIT in a MASH model. VWR alone had no significant impact on MFN2 protein expression [18,21]. Furthermore, in the study by da Costa Fernandes et al. [16] RE was found to inhibit Mfn2 mRNA expression, contrasting with the disease-induced upregulation observed in their specific model.

OPA1 expression is typically reduced in MASLD/MASH models [20,21,22,23], although some studies reported no significant changes [18,26]. The regulation of OPA1 by exercise in rodent models exhibits intensity dependence and heterogeneity. A number of studies have indicated that MICT and VWR do not induce significant alterations in OPA1 expression [18,20,21]. Li et al. [26] demonstrated that MICT exerted no appreciable effect, whereas HIIT markedly elevated OPA1 protein levels. Hu et al. [22] found that combined training also increased OPA1 protein. In a zebrafish model, MICT was sufficient to promote OPA1 protein expression [23].

In summary, MASLD/MASH pathology leads to the downregulation of the fusion proteins MFN1 and OPA1. The expression of MFN2 appears to be disease-stage dependent, being downregulated in MASLD but potentially unchanged in MASH. Aerobic exercise, including MICT and HIIT, is the most effective intervention for restoring fusion markers. Notably, the improvement of OPA1 may be contingent upon high-intensity exercise. In contrast, VWR exerts a weaker regulatory effect on fusion proteins, while resistance training may inhibit the expression of fusion genes under specific conditions.

**Table 1:** Effects of Exercise Interventions on Mitochondrial Dynamics-Related Proteins.

Reference	Study Model			Study Protocol			Key Results ↓ Down Significantly, ↑ Up Significantly, ↔ No Significant	
	Specie Sex	Age	Disease Stage	Modeling Protocol	Exercise Classification	Exercise Protocol Details	MASLD/MASH vs. Control	Exercise vs. MASLD/MASH
Zou et al. (2023) [23]	Zebrafish AB strain	6 months	MASLD	12 weeks HFD (24% energy from fat) Exercise concurrent with HFD	Aerobic Exercise MICT	Mode: Swimming Intensity: Month 1: 6 × BL/s (~40% Ucrit) for 4 h Months 2–3: 8 × BL/s (~55% Ucrit) for 4 h Freq: 5 d/w Duration: 12 weeks	MASLD: ↓ MFN2 protein ↓ OPA1 protein ↓ Drp1 protein	MICT: ↑ MFN2 protein ↔ OPA1 protein ↑ Drp1 protein
Hu et al. (2023) [22]	Rats Sprague-Dawley Male	4–5 weeks	MASLD	11 weeks HFD (60% energy from fat) Exercise started at week 6 of HFD	Combined Exercise RE + MICT	Mode: Treadmill Intensity: Resistance: 10–25° incline, 20–25 cm/s (2 min)/rest (1 min) × 8 cycles Aerobic: Continuous running 30 min Freq: 5 d/w Duration: 5 weeks	MASLD: ↓ MFN2 protein ↓ OPA1 protein ↑ Drp1 protein	RE + MICT: ↑ MFN2 protein ↑ OPA1 protein ↓ Drp1 protein
Deng et al. (2025) [17]	Rats Sprague-Dawley Male	300 g	MASLD	16 weeks HFD (60% energy from fat) Exercise started at week 8 of HFD	Aerobic Exercise HIIT or MICT	Mode: Treadmill Intensity: HIIT: 85–90% Smax (4 min)/50–60% Smax (2 min) × 10 cycles MICT: 70% Smax for 60 min Freq: 7 d/w Duration: 8 weeks Volume-matched HIIT and MICT	MASLD: ↓ MFN1 protein ↓ MFN2 protein ↑ Fis1 protein	HIIT: ↑ MFN1 protein ↑ MFN2 protein ↓ Fis1 protein MICT: ↑ MFN1 protein ↑ MFN2 protein ↓ Fis1 protein
Gonçalves et al. (2016) [18]	Rats Sprague-Dawley Male	6–7 weeks	MASH	17 weeks liquid HFD (71% energy from fat) VWR concurrent with HFD, MICT started at week 9 of HFD	Aerobic Exercise MICT or VWR	Mode: Treadmill (MICT) or Voluntary wheel (VWR) MICT: Intensity: 15–25 m/min for 60 min Freq: 5 d/w Duration: 8 weeks VWR: Voluntary wheel running with ad libitum Duration: 17 weeks	MASH: ↓ MFN1 protein ↔ MFN2 protein ↔ OPA1 protein ↔ Drp1 protein	MICT: ↑ MFN1 protein ↑ MFN2 protein ↔ OPA1 protein ↔ Drp1 protein VWR: ↔ MFN1 protein ↔ MFN2 protein ↔ OPA1 protein ↔ Drp1 protein
Andani et al. (2024) [25]	Rats Wistar Male	230 ± 10 g	MASH	DEX injected at week 8 (2.5 → 10 mg/kg) Exercise performed prior/during	Aerobic Exercise HIIT or MICT	Mode: Treadmill Intensity: HIIT: 5% incline, 85% VO_2_peak (40 m/min, 3 min)/20 m/min (3 min) × 6 cycles MICT: 0 incline, 20 m/min Freq: 3 d/w Duration: 8 weeks Volume-matched HIIT and MICT	MASH: ↔ MFN2 mRNA	HIIT: ↔ MFN2 mRNA MICT: ↔ MFN2 mRNA
Stevanović-Silva et al. (2023) [20]	Rats Sprague-Dawley Female	7 weeks	GDM MASLD	18 weeks HFHS (42% energy from fat containing high cholesterol and 31% energy from carbohydrates mainly as sucrose) Exercise during pregnancy	Aerobic Exercise MICT + VWR	Mode: Treadmill + Voluntary Wheel MICT: Intensity: Week1: 18 m/min for 20–60 min, Weeks 2–3: 21 m/min for 60 min Freq: 6 d/w Duration: 3 weeks VWR: Voluntary wheel running ad libitum	GDM MASLD: ↔ MFN1 protein ↓ MFN2 protein ↓ OPA1 protein ↔ Drp1 protein (trend up) ↔ MFN1 mRNA ↔ MFN2 mRNA (trend down) ↔ Drp1 mRNA (trend down)	MICT + VWR: ↑ MFN1 protein ↑ MFN2 protein ↔ OPA1 protein ↓ Drp1 protein ↔ MFN1 mRNA ↔ MFN2 mRNA (trend up) ↔ Drp1 mRNA (trend up)
Li et al. (2024) [26]	Mice C57BL/6J Male	12 weeks	MASLD	8 weeks HFD (45% energy from fat) Exercise concurrent with HFD	Aerobic Exercise HIIT or MICT	Mode: Treadmill Intensity: HIIT: 85% Smax (2 min)/40% HIS (2 min) × 12 cycles MICT: 45–50% Smax for 60 min Freq: 5 d/w Duration: 8 weeks HIS every week increase 1 m/min, Volume-matched HIIT and MICT	MASLD: ↔ OPA1 protein ↑ Fis1 protein	HIIT: ↑ OPA1 protein ↓ Fis1 protein MICT: ↔ OPA1 protein ↓ Fis1 protein
Rosa-Caldwell et al. (2017) [21]	Mice C57BL/6J Male	8 weeks	MASLD	8 weeks WD (42% energy from fat containing 1.5 g/kg cholesterol) Exercise started at week 4 of WD	Aerobic Exercise VWR	Voluntary wheel running ad libitum Duration: 4 weeks	MASLD: ↔ MFN1 mRNA ↔ MFN2 mRNA ↓ OPA1 mRNA ↓ MFN2 protein ↓ Mff mRNA ↓ Drp1 mRNA ↔ Fis1 mRNA ↓ Drp1 protein	VWR: ↔ MFN1 mRNA ↔ OPA1 mRNA ↑ MFN2 mRNA ↔ MFN2 protein ↔ Mff mRNA ↔ Drp1 mRNA ↔ Fis1 mRNA ↔ Drp1 protein
da Costa Fernandes et al. (2025) [16]	Mice Swiss Male	8 weeks	MASLD	14 weeks HFD (60% energy from fat) Exercise after model establishment	Resistance Exercise	Mode: Ladder Climbing Intensity: 70% MVCC/rest (60–90 s) × 20 climbs Freq: 7 d/w Duration: 8 weeks	MASLD: ↓ MFN1 mRNA ↑ MFN2 mRNA ↑ Fis1 mRNA ↑ Drp1 mRNA	RE: ↓ MFN1 mRNA ↓ MFN2 mRNA ↓ Fis1 mRNA ↓ Drp1 mRNA
Bórquez et al. (2024) [24]	Mice C57BL/6J Male	4 weeks	MASLD	12 weeks HFD (60% energy from fat) Exercise started at week 8 of HFD	Aerobic Exercise MICT	Mode: Treadmill Intensity: 60–65% Smax for 60 min Freq: 5 d/w Duration:4 weeks	MASLD: ↔ MFN2 protein	MICT: ↑ MFN2 protein
Bórquez et al. (2024) [24]	Mice C57BL/6J Male	8 weeks	MASH	4 weeks MCD (methionine choline-deficient diet combined 45% HFD) Exercise concurrent with MCD	Aerobic Exercise MICT	Mode: Treadmill Intensity: 60–65% Smax for 60 min Freq: 5 d/w Duration:4 weeks	MASH: ↔ MFN2 protein	MICT: ↑ MFN2 protein
Wang et al. (2023) [19]	Mice C57BL/6J Male	6 weeks	T2DM MASH	20 weeks HFD (60% energy from fat) STZ injected at week 12 of HFD Exercise started at week 12 of HFD	Aerobic Exercise HIIT	Mode: Treadmill Intensity: 15° incline, 16 m/min (4 min)/rest (2 min) × 12 cycles Freq: 5 d/w Duration: 8 weeks Weeks 1–4 speed increased by 2 m/min every week Weeks 5–8 speed increased by 1 m/min every week	T2DM MASH: ↓ MFN1 mRNA ↓ MFN2 mRNA ↔ Drp1 mRNA	HIIT: ↔ MFN1 mRNA ↑ MFN2 mRNA ↓ Drp1 mRNA

In this study, the MASLD group refers to subjects with simple steatosis, distinct from the MASH group. ↑: upregulated; ↓: downregulated; ↔: no significant change. Abbreviations: DEX, Dexamethasone; Drp1, Dynamin-related protein 1; Fis1, Fission protein 1; GDM, Gestational Diabetes Mellitus; HFD, High-Fat Diet; HFHS, High-Fat High-Sucrose diet; HIIT, High-Intensity Interval Training; HIS, High-Intensity Speed; MASLD, Metabolic dysfunction-associated steatotic liver disease; MASH, Metabolic dysfunction-associated steatohepatitis; MCD, Methionine-Choline Deficient diet; Mff, Mitochondrial fission factor; MFN1/2, Mitofusin 1/2; MICT, Moderate-Intensity Continuous Training; MVCC, Maximal Voluntary Carrying Capacity; OPA1, Optic Atrophy 1; RE, Resistance Exercise; Smax, Speed at exhaustion; STZ, Streptozotocin; T2DM, Type 2 Diabetes Mellitus; Ucrit, Critical swimming speed; VO_2_peak, Peak oxygen uptake; VWR, Voluntary Wheel Running.

#### 3.3.2. Effects of Exercise on Mitochondrial Fission Proteins

Mitochondrial fission is primarily mediated by Drp1, Fis1, and mitochondrial fission factor (Mff), with Drp1 and Fis1 being the focal points of research. Unlike the relatively consistent downregulation of fusion proteins, alterations in fission proteins during MASLD/MASH present a complex pattern.

Drp1 exhibits high heterogeneity in disease models. Zou et al. [23] and Rosa-Caldwell et al. [21] found decreased Drp1 protein in MASLD, whereas da Costa Fernandes et al. [16] and Hu et al. [22] reported elevated expression. No significant changes were observed in other MASH or GDM-MASLD models [18,19,20]. This heterogeneity in baseline expression dictates the directional outcomes of exercise interventions. In models where Drp1 may be overexpressed or unchanged, high-intensity or greater exercise volume has been observed to reduce its expression. Combined training, HIIT, and RE each led to a significant decrease in Drp1 protein expression [16,19,22]. Stevanović-Silva et al. [20] found that MICT combined with daily VWR reduced Drp1 protein, despite an opposing trend in mRNA. It appears that low-intensity exercise is inadequate in inducing substantial changes in Drp1. Neither MICT nor VWR have been shown to induce significant protein alterations [18,21]. However, in a zebrafish model, moderate-intensity swimming significantly increased Drp1 protein expression [23].

In contrast to Drp1, Fis1 shows a consistent trend. HFD interventions consistently led to significantly increased Fis1 expression [16,17,26]. Exercise interventions targeting Fis1 were highly effective and consistent. MICT, HIIT, and RE all significantly downregulated Fis1 expression [16,17,26]. However, VWR failed to induce significant changes in Fis1 mRNA in a WD-induced model [21]. Data on Mff is scarce; only Rosa-Caldwell et al. [21] reported a significant decrease in Mff mRNA in MASLD, which was unaffected by VWR.

In summary, the imbalance of mitochondrial fission in MASLD/MASH is characterized by a consistent elevation of Fis1. Exercise interventions primarily inhibit excessive pathological fission by downregulating Fis1. The regulation of Drp1 appears to be dependent on exercise intensity and volume. Notably, resistance training demonstrates efficacy similar to other modalities in inhibiting fission proteins, contrasting with its unique suppressive profile in fusion protein regulation.

### 3.4. Regulatory Effects of Exercise on Mitochondrial Quality Control

Beyond mitochondrial dynamics, MQC encompasses mitochondrial biogenesis and mitophagy. Nine of the included studies concurrently measured markers for both processes (Table 2).

#### 3.4.1. Effects of Exercise on Mitochondrial Biogenesis

Most studies assessed biogenesis via peroxisome proliferator-activated receptor gamma coactivator 1-alpha (PGC-1α) and mitochondrial transcription factor A (TFAM) with fewer measuring nuclear respiratory factor 1/2 (NRF1/2). In the majority of models [17,18,19,21,23], protein or mRNA levels of biogenesis markers were significantly reduced. Stevanović-Silva et al. [20] found no significant changes in these proteins in a GDM-associated MASLD model. Conversely, da Costa Fernandes et al. [16] reported that HFD intervention paradoxically led to significant increases in PGC-1α protein and NRF2 mRNA/protein.

Exercise modalities exert divergent effects on biogenesis. MICT and HIIT showed consistent efficacy; multiple studies confirmed that both modalities significantly induced the expression of PGC-1α and TFAM [17,18,19,20,23]. The effects of VWR were inconsistent, with Gonçalves et al. [18] reporting increased PGC-1α protein and Rosa-Caldwell et al. [21] finding no change in mRNA. Notably, RE exhibited a potential inhibitory effect; da Costa Fernandes et al. [16] showed that RE significantly decreased the expression of PGC-1α, TFAM, and NRF1/2.

#### 3.4.2. Effects of Exercise on Mitophagy and Autophagic Flux

Mitophagy was assessed via the PTEN-induced kinase 1 (PINK1)/E3 ubiquitin-protein ligase Parkin (Parkin) pathway and flux markers. In MASLD/MASH models, the suppression of the canonical PINK1/Parkin pathway (reduced protein expression) was the most consistent finding [17,18,20,23,26]. Aerobic exercise demonstrated strong consistency in restoring PINK1/Parkin expression; most MICT and HIIT protocols significantly increased their levels [17,18,20,23,26], whereas VWR did not [18]. In the GDM-associated model, Stevanović-Silva et al. [20] observed that MICT failed to reverse the reduction in Parkin.

Regarding autophagic flux, baseline alterations varied across models. Zou et al. [23] and Li et al. [26] observed elevated sequestosome-1 (P62) protein or increased Microtubule-associated protein 1 light chain 3 (LC3)-II/LC3-I ratios in MASLD, suggesting blocked flux. However, other studies found no significant changes [18,20,21]. In terms of intervention, moderate-to-low intensity exercise tended to unclog autophagic flux. MICT significantly reduced accumulated P62 or elevated LC3 ratios in MASLD models [23,26], though it had no significant effect in a MASH model [18]. In the WD-induced MASLD model, Rosa-Caldwell et al. [21] found that VWR significantly increased the LC3-II/LC3-I ratio and decreased P62, whereas VWR had no effect in the MASH model [18]. HIIT showed no significant impact on the LC3-II/LC3-I ratio in one MASLD study [26].

Additionally, Li et al. [26] found that MASLD led to elevated BCL2/adenovirus E1B 19 kDa protein-interacting protein 3 (BNIP3) protein, which was significantly downregulated by both MICT and HIIT. Conversely, Rosa-Caldwell et al. [21] reported decreased BNIP3 protein in MASLD, which was unaffected by VWR. MICT was also found to significantly increase damage-regulated autophagy modulator (DRAM) mRNA expression in a MASH model by Andani et al. [25].

In summary, in MASLD/MASH models, the canonical mitophagy pathway (PINK1/Parkin) is consistently suppressed. HIIT and MICT show strong consistency in restoring PINK1 and Parkin expression. While baseline changes in autophagic flux markers vary across models, exercise generally tends to facilitate the unclogging or enhancement of autophagic flux.

**Figure 3:**
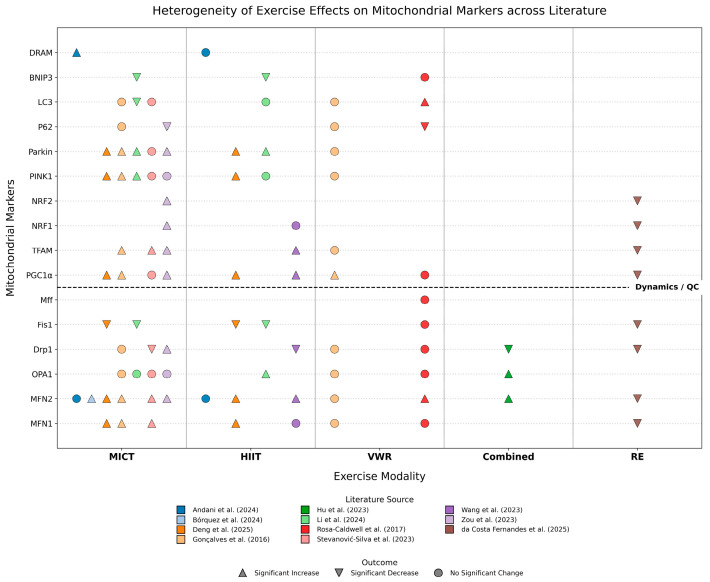
Molecular Landscape of Exercise-Induced Regulation on Hepatic Mitochondrial Quality Control Networks in MASLD/MASH Models [16,17,18,19,20,21,22,23,24,25,26]. The Upward Triangle (△) indicates a Significant Increase; the Downward Triangle (▽) indicates a Significant Decrease; the Circle (○) indicates No Significant Change. Abbreviations: MICT, Moderate-Intensity Continuous Training; HIIT, High-Intensity Interval Training; VWR, Voluntary Wheel Running; Combined, Combined aerobic and resistance training; RE, Resistance Training; MFN, Mitofusin; OPA1, Optic Atrophy 1; Drp1, Dynamin-related protein 1; Fis1, Mitochondrial fission 1 protein; Mff, Mitochondrial fission factor; PGC-1α, Peroxisome proliferator-activated receptor gamma coactivator 1-alpha; TFAM, Mitochondrial transcription factor A; NRF1, Nuclear respiratory factor 1; NRF2, Nuclear respiratory factor 2; Parkin, E3 ubiquitin-protein ligase Parkin; PINK1, PTEN-induced kinase 1; LC3, Microtubule-associated protein 1 light chain 3; BNIP3, BCL2/adenovirus E1B 19 kDa protein-interacting protein 3; DRAM, Damage-regulated autophagy modulator.

To systematically summarize and compare the regulatory patterns of different exercise interventions on hepatic mitochondrial indices, we integrated results from all 11 included studies to cross-compare the effects of five exercise modalities—MICT, HIIT, VWR, combined training, and RE—on the expression of 16 key molecules involved in mitochondrial fusion, fission, biogenesis, and quality control (Figure 3). The overall analysis reveals that the effects of exercise interventions exhibit significant “marker specificity” and “modality dependence.”

Specifically, three distinct regulatory characteristics can be identified from the landscape: First, consistency in improvement: Regardless of the exercise modality, the suppression of Fis1 overexpression and the upregulation of MFN2 appear as the most universal beneficial mechanisms, highlighting the core commonality of exercise in restoring mitochondrial network connectivity and inhibiting pathological fragmentation. Second, heterogeneity in regulation: The alteration of the fission protein Drp1 exhibits drastic fluctuations across studies (increase, decrease, or no change). This high degree of heterogeneity visually reflects the sensitive dependence of this marker on the stage of disease progression and the specific intervention protocol. Finally, modality specificity: Aerobic exercise (MICT and HIIT) demonstrates broad and stable positive regulatory effects on activating biogenesis (PGC-1α, TFAM) and restoring mitophagy pathways (PINK1/Parkin). In contrast, resistance training and voluntary wheel running display unique molecular fingerprints, such as the potential suppression of certain fusion genes by resistance training and the ineffectiveness of voluntary exercise on specific markers. This visual summary compellingly suggests that while exercise is generally beneficial, the remodeling of its molecular targets is highly dependent on the type and intensity of the exercise.

## 4. Discussion

### 4.1. Dysregulated Mitochondrial Dynamics in MASLD Pathogenesis

**Figure 4:**
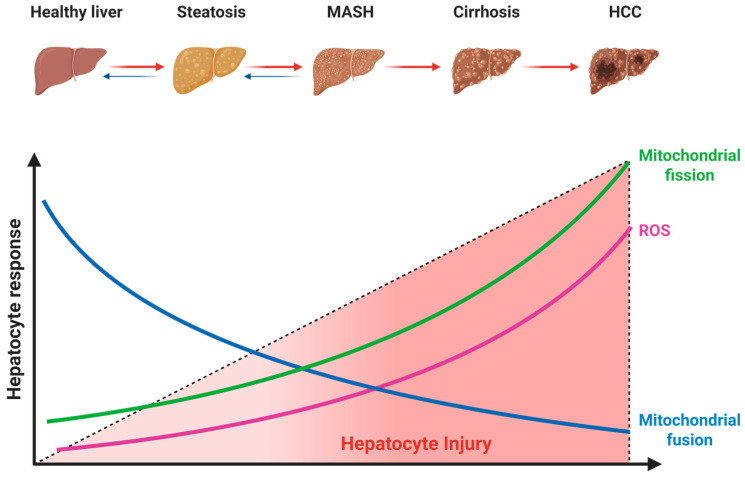
Alterations in mitochondrial dynamics during MASLD progression. The dashed line represents the increasing severity of hepatocyte injury. Red arrows denote the progression of liver disease, and blue arrows indicate the potential for reversibility, particularly in early stages. Abbreviations: MASH, metabolic dysfunction-associated steatohepatitis; HCC, hepatocellular carcinoma. Created with BioRender.com (accessed on 15 November 2025).

The liver is densely populated with mitochondria to sustain its high metabolic demand. The maintenance of this pool relies on mitochondrial dynamics, a continuous cycle of fusion and fission that governs mitochondrial morphology, network plasticity, and spatial distribution [27,28]. In MASLD, this dynamic equilibrium is severely disrupted, shifting towards a fragmented phenotype that compromises oxidative phosphorylation efficiency and elicits the release of proinflammatory cytokines [29] (Figure 4).

#### 4.1.1. Suppression of Mitochondrial Fusion

**Figure 5:**
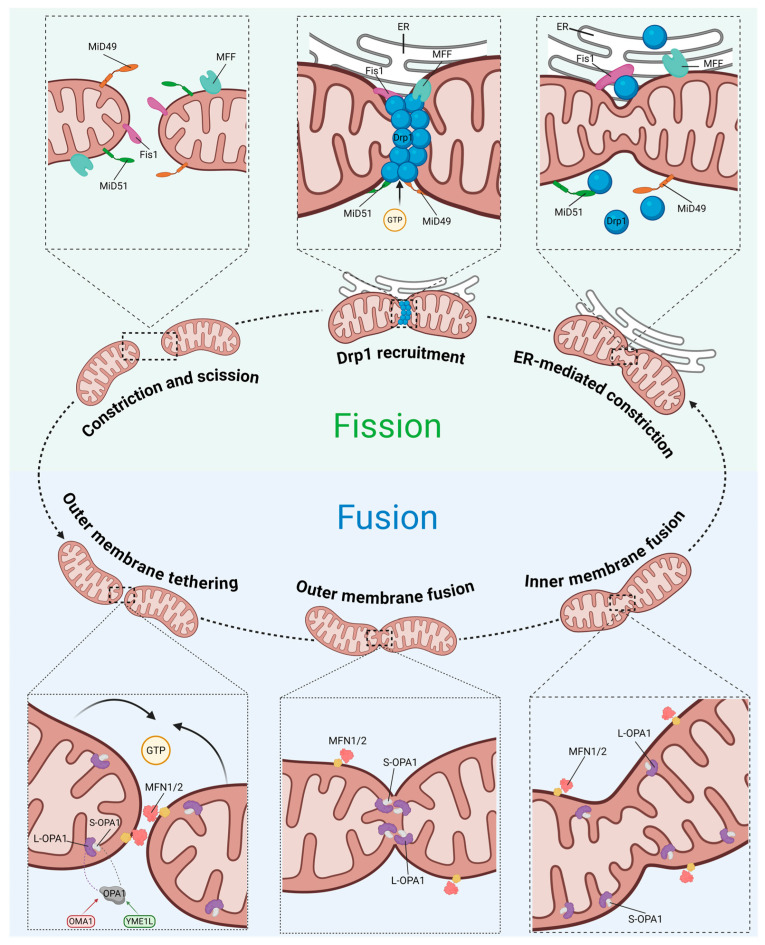
Schematic of mitochondrial fusion and fission machinery. Abbreviations: MiD49, mitochondrial dynamics protein of 49 kDa; MiD51, mitochondrial dynamics protein of 51 kDa; Fis1, fission protein 1; MFF, mitochondrial fission factor; Drp1, dynamin-related protein 1; MFN1/2, mitofusin 1/2; OPA1, optic atrophy 1; OMA1, mitochondrial inner membrane zinc-dependent metalloprotease; YME1L, yeast mitochondrial escape 1-like ATPase. Created with BioRender.com (accessed on 15 November 2025).

Mitochondrial fusion facilitates the homogenization of trans-inner membrane potential, mitochondrial DNA (mtDNA) complementation, and the sharing of tricarboxylic acid cycle intermediate pools by merging adjacent mitochondria. This fusion machinery is primarily orchestrated by key regulators, including MFN1 and MFN2 anchored to the outer mitochondrial membrane, and OPA1 residing in the inner membrane [30] (Figure 5).

Beyond its pivotal role in mitochondrial fusion, MFN2 also tethers mitochondria to the endoplasmic reticulum (ER), facilitating lipid transfer and insulin signaling to maintain energy homeostasis [31]. However, in the context of MASLD, MFN2 expression is significantly downregulated [32,33]. This downregulation compromises the integrity of ER-mitochondria contact sites, impairs mitochondrial membrane phosphatidylethanolamine biosynthesis, and reduces oxidative phosphorylation efficiency, thereby exacerbating intracellular lipid accumulation [32]. Furthermore, MFN2 suppression triggers robust ER stress and inflammatory responses, driving the pathological progression from steatosis to fibrosis and hepatocellular carcinoma [34]. Similarly, MFN1 is markedly downregulated in steatohepatitis models, an effect further exacerbated by proinflammatory cytokines [35].

Within mitochondria, OPA1 preserves inner membrane fusion and the tightness of cristae junctions, a function critical for sequestering pro-apoptotic factors such as cytochrome c within the cristae lumen to prevent their release and for optimizing the assembly of respiratory supercomplexes [36,37]. The functionality of OPA1 hinges on a precise stoichiometric balance between its long and short isoforms, a dynamic equilibrium governed by the proteases Mitochondrial inner membrane zinc-dependent metalloprotease (OMA1) and Yeast mitochondrial escape 1-like ATPase (YME1L) [38]. In MASLD, this balance is frequently disrupted [22,23]. The consequent OPA1 dysfunction not only induces excessive mitochondrial fragmentation but also dismantles cristae architecture, impairs ATP synthesis, and heightens susceptibility to apoptosis, thereby constituting a core driver of hepatocyte injury [39].

#### 4.1.2. Aberrant Activation of Mitochondrial Fission

The central regulator governing mitochondrial fission is Drp1, encoded by the DNM1L gene in humans [40]. Structurally, Drp1 lacks a pleckstrin homology domain capable of directly recognizing and binding lipid membranes. Consequently, its recruitment from the cytosol to prospective fission sites on the outer mitochondrial membrane relies on specific receptors, such as MFF, Fis1, and MiD49/51, to initiate the subsequent membrane constriction and scission [40] (Figure 5).

In sharp contrast to the suppression of mitochondrial fusion, mitochondrial fission processes are aberrantly hyperactivated in MASLD [22,23,41]. The aberrant activation of Drp1 exacerbates hepatic lipid metabolic imbalance through multiple pathways. It not only disrupts mitochondrial homeostasis, leading to diminished ATP synthesis efficiency and cellular energy metabolic defects [42], but also reprograms lipid metabolism towards a pathological state characterized by “increased synthesis and decreased lipolysis” [43]. Furthermore, aberrant Drp1 activation precipitates functional defects in succinate dehydrogenase, thereby interfering with tricarboxylic acid cycle flux and insulin signal transduction, which further promotes abnormal hepatic lipid accumulation [44,45]. Ultimately, the excessive ROS generated by mitochondrial fragmentation trigger oxidative stress and inflammatory cascades, establishing a vicious cycle where metabolic dysregulation and inflammatory injury mutually reinforce one another [42].

Notably, the role of Drp1 in MASLD is characterized by distinct stage-specificity. During the early phase of hepatic steatosis, Drp1 hyperactivation serves as a pivotal pathogenic driver. Consequently, inhibiting its activity can effectively ameliorate mitochondrial dynamic imbalances and attenuate the progression of lipid deposition in hepatocytes [41,46,47]. Conversely, as the disease progresses to MASH, the functional role of Drp1 shifts, becoming indispensable for maintaining mitochondrial quality control. At this stage, Drp1 is critical for the clearance of dysfunctional mitochondria; excessive inhibition leads to the accumulation of damaged organelles, thereby exacerbating cellular stress, inflammatory responses, and the overexpression of profibrotic genes [48]. Therefore, therapeutic strategies targeting Drp1 must be precisely tailored to the stage of disease progression. In advanced stages, simplistic ‘one-size-fits-all’ inhibitory approaches may yield counterproductive outcomes.

### 4.2. Effects of Exercise on Mitochondrial Dynamics

**Figure 6:**
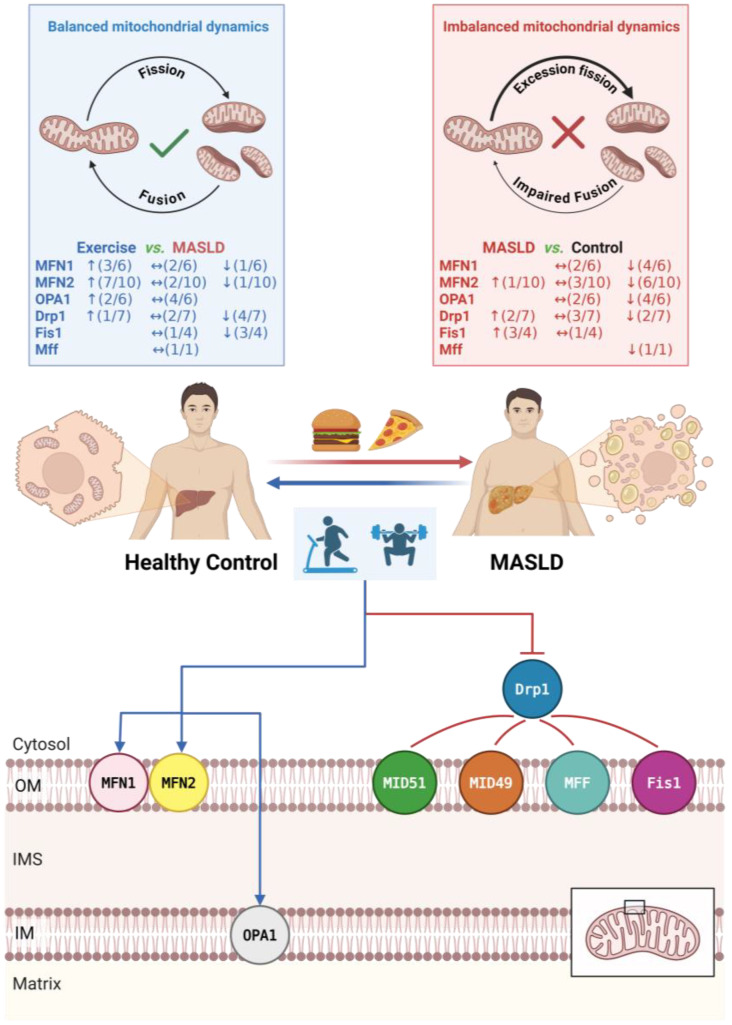
Proposed mechanisms of exercise intervention on mitochondrial dynamics in MASLD. Arrows indicate the direction of change (↑: upregulated; ↓: downregulated; ↔: no significant change). Numbers in parentheses denote the number of studies reporting that specific outcome out of the total number of studies assessing that marker. The black box indicates the region of the mitochondrial membrane structure shown in the detailed schematic. Abbreviations: Drp1, dynamin-related protein 1; Fis1, fission protein 1; MFF, mitochondrial fission factor; MFN1/2, mitofusin 1/2; MiD49/51, mitochondrial dynamics proteins of 49/51 kDa; OPA1, optic atrophy 1. Created with BioRender.com (accessed on 15 November 2025).

The present study aimed to explore the effects of different exercise modalities on hepatic mitochondrial dynamics in MASLD/MASH models. We found that different exercise interventions exhibited distinct, modality-specific regulatory patterns [49]: while aerobic exercise (MICT and HIIT) served as the primary driver for restoring mitochondrial network homeostasis, the remodeling of specific proteins was intensity-dependent, whereas resistance training and voluntary exercise displayed unique, and occasionally divergent, molecular effects (Figure 6).

#### 4.2.1. Effects on Fusion Proteins

Our analysis reveals that MFN2 is the most sensitive target of hepatic mitochondrial fusion in response to exercise. In various MASLD/MASH models, MFN2 is significantly downregulated, and aerobic exercise can significantly upregulate its expression via PGC-1α-dependent pathways [18,19]. In contrast, the restoration of the inner membrane fusion protein OPA1 exhibits significant “intensity dependence”. Li et al. [26] noted that only HIIT significantly increased OPA1 protein levels, whereas MICT was ineffective. The mechanism behind this phenomenon may relate to the activation threshold of the cellular energy sensor AMP-activated protein kinase (AMPK). High-level mechanistic studies indicate that AMPK can directly phosphorylate OPA1 or regulate mitochondrial membrane potential to maintain cristae tightness [50]. The high metabolic stress generated by HIIT may more effectively activate this pathway, thereby specifically driving OPA1-mediated inner membrane remodeling.

Notably, RE exhibits a unique pattern of impact on fusion proteins. da Costa Fernandes et al. [16] found that while RE increased bioenergetic markers such as ATP synthase subunit alpha (ATP5), it suppressed MFN1 mRNA expression. This may be linked to the competitive inhibition between the mTORC1 signaling pathway activated by RE and the PGC-1α-driven biogenesis pathway in the liver [51], suggesting potential limitations of resistance training alone in improving hepatic mitochondrial morphology.

#### 4.2.2. Effects on Fission Proteins

Drp1 exhibits high model-dependent heterogeneity in MASLD/MASH. First, in rodent models where Drp1 is overactivated or even unchanged, exercise tends to decrease its expression to inhibit pathological fission. For instance, both RE and combined training significantly reduced elevated Drp1 levels [16,22]. Wang et al. [19] found that in T2DM combined with MASH models, although baseline Drp1 changes were not significant, HIIT still induced a significant downregulation of Drp1. The molecular mechanism underlying this inhibitory effect has been elucidated by Hu et al. [22]: exercise activates the nicotinamide adenine dinucleotide (NAD)+-dependent deacetylase Sirt1, which directly deacetylates Drp1 at the K642 site, thereby inhibiting its translocation to mitochondria and excessive fission activity. Furthermore, da Costa Fernandes et al. [16] discovered that the suppression of Drp1 by RE might be linked to its epigenetic regulation of MTCH2 (Mitochondrial Carrier Homolog 2) promoter methylation, subsequently remodeling the metabolic environment of the outer mitochondrial membrane.

Second, the regulation of Drp1 appears to exhibit a distinct “intensity threshold.” While HIIT significantly downregulated pathologically elevated Drp1 levels, lower-intensity interventions such as VWR and standalone MICT failed to induce significant changes in Drp1 protein expression. However, augmenting the exercise load proved effective [18,21]. Stevanović-Silva et al. [20] found that combining MICT with daily VWR significantly reduced Drp1 protein, a beneficial effect also observed with combined aerobic and resistance training strategies. Notably, in a zebrafish model, Zou et al. [23] reported that MICT conversely led to an increase in Drp1 protein expression. This discrepancy may reflect an adaptive response specific to lower vertebrates or early disease stages, where a moderate increase in fission facilitates necessary mitochondrial turnover and biogenesis.

Unlike the heterogeneity of Drp1, another fission receptor protein, Fis1, exhibits high consistency. HFD typically leads to a significant increase in Fis1 expression, indicating a generalized activation of mitochondrial fission mechanisms in pathological states [16,17]. Various forms of exercise interventions (MICT, HIIT, and RE) consistently and significantly downregulate Fis1 expression, effectively curbing excessive mitochondrial fission. Regarding Mff, Rosa-Caldwell et al. [21] reported that its mRNA was decreased in a WD model and insensitive to voluntary exercise.

In summary, the exercise-mediated regulation of mitochondrial fission is multidimensional: it inhibits pathologically activated Drp1 and Fis1 through Sirt1-mediated deacetylation and epigenetic modifications, but in specific contexts (such as adaptive compensation phases), it may also maintain or fine-tune Drp1 levels to coordinate with biogenesis, a process highly dependent on sufficient exercise intensity or load.

Based on the synthesized evidence, we propose the existence of a potential “intensity threshold” governing hepatic mitochondrial dynamics. Although direct comparisons within the same study are limited, a cross-study analysis suggests a distinct pattern: the restoration of MFN2-mediated outer membrane fusion and the suppression of aberrant Fis1 activity appear achievable via MICT. In contrast, profound structural remodeling of the inner membrane (OPA1) and the reversal of pathological Drp1 activation seem to necessitate higher-intensity stimuli (e.g., HIIT) or a greater cumulative exercise load. Furthermore, future investigations should focus on post-translational modifications (e.g., acetylation, SUMOylation) to bridge the gap between transcriptional profiles and protein abundance.

### 4.3. Effects of Exercise on Mitochondrial Quality Control Indicators

#### 4.3.1. Effects on Mitochondrial Biogenesis

In most studies, exercise-induced dynamic remodeling is highly synchronized with the restoration of mitochondrial biogenesis. Both MICT and HIIT can significantly upregulate PGC-1α and its downstream transcription factor TFAM [19,23]. The mechanism of this synergistic effect lies in PGC-1α acting not only as the “master regulator” of biogenesis but also as a transcriptional co-activator for MFN2, ensuring that newly generated mitochondria can rapidly fuse into the network. However, da Costa Fernandes et al. [16] pointed out the distinctiveness of RE, which showed a suppressive trend for PGC-1α mRNA. This suggests that when prescribing exercise for MASH patients with sarcopenia, caution is needed regarding the potential interference of resistance training alone on hepatic mitochondrial turnover.

#### 4.3.2. Effects on Mitophagy

Exercise plays a crucial “scavenger” role in regulating mitophagy. Rosa-Caldwell et al. [16] found that even low-intensity VWR significantly enhanced basal autophagic flux (increased LC3-II/I, decreased p62). This suggests that enhancing autophagy may be one of the earliest adaptive responses of exercise improving liver health. Mechanistically, HIIT showed stronger consistency than MICT in restoring the PINK1/Parkin pathway [26], which may be related to high-intensity exercise more effectively inducing transient mitochondrial depolarization, thereby triggering the stabilization of PINK1 on the outer mitochondrial membrane [52]. Additionally, Andani et al. [25] found that MICT specifically upregulated DRAM, indicating that different exercise modalities may activate distinct autophagy receptor pathways to clear damaged mitochondria.

Ultimately, the orchestrated restoration of mitochondrial dynamics and quality control machinery by exercise is not an end in itself, but a means to re-establish hepatic bioenergetic homeostasis. The exercise-induced restoration of mitochondrial dynamics proteins represents more than a mere morphological adaptation; it functionally translates into enhanced hepatic metabolic capacity. Specifically, the upregulation of fusion proteins extends beyond facilitating outer membrane fusion; it plays a pivotal role in reconstituting the physical tethers at mitochondria-associated endoplasmic reticulum membranes (MAMs) and lipid droplet-mitochondria contact sites [24,53]. The re-establishment of these organelle interfaces is indispensable for optimizing calcium signaling and facilitating the direct transfer of fatty acids into the mitochondrial matrix, thereby potentiating β-oxidation rates, maximizing ATP production, and suppressing lipotoxicity-induced apoptosis [24,54]. Concurrently, the exercise-mediated suppression of pathological fission directly alleviates reactive oxygen species (ROS) generation and mitochondrial depolarization, which functionally manifests as improved hepatic insulin sensitivity and augmented whole-body fat oxidation [22,55]. Together, these molecular adaptations forge a robust link between mitochondrial structural remodeling and the clinical alleviation of hepatic steatosis and inflammation.

### 4.4. Limitations

Several limitations in the current body of literature must be acknowledged. The literature search was restricted to PubMed and Web of Science due to institutional subscription limitations; although these are major resources, this constraint might have missed studies indexed elsewhere, resulting in a relatively modest final sample size (*n* = 11). The substantial heterogeneity across these studies regarding animal species, diet protocols, comorbidities, and exercise modalities precluded the feasibility of a quantitative meta-analysis. In terms of biological variables, a distinct sex bias was evident, with the vast majority of studies utilizing male animals, likely to minimize estrous cycle-related confounding. However, this predominance prevents the drawing of definitive conclusions regarding sex-specific mitochondrial responses to exercise, which is a critical gap given the significant sexual dimorphism often observed in metabolic diseases. Methodologically, most findings rely on static markers (e.g., Western blot, qPCR) rather than dynamic functional assays in live cells. Furthermore, regarding study quality, domains such as blinding and allocation concealment were frequently assessed as high or unclear risk. While the lack of blinding is an intrinsic challenge in animal exercise research and partially mitigated by the use of objective biochemical outcomes, these methodological limitations suggest that the strength of the current evidence should be interpreted with appropriate caution. Moreover, the majority of analyzed models reflect early-to-intermediate disease stages (steatosis or steatohepatitis) with interventions that were often short-term and applied preventively or concurrently. Consequently, evidence regarding the therapeutic efficacy of exercise in reversing established, advanced fibrosis or cirrhosis remains scarce. This lack of data on advanced disease stages, combined with inherent interspecies differences, limits the direct translational relevance of current findings to human clinical practice.

## 5. Conclusions

In conclusion, this systematic review demonstrates that, in animal models, exercise serves as a pivotal physiological strategy for ameliorating mitochondrial dysregulation associated with MASLD and its progression. Exercise exerts beneficial effects on hepatic mitochondrial network homeostasis by synergistically coordinating the fusion–fission equilibrium, mitochondrial biogenesis, and autophagic processes. Current evidence points to potential characteristics of “modality specificity” and “intensity dependence” in these remodeling effects. While aerobic exercise appears to act as a consistent driver for functional restoration, profound structural remodeling may be contingent upon a specific “intensity threshold.” Conversely, resistance training seems to display a distinct molecular regulatory profile. Although current evidence is primarily derived from rodent models and exhibits heterogeneity, the highly conserved nature of mitochondrial quality control machinery across mammals renders this study an important theoretical foundation for future research. To advance translational research from mechanism to clinical application, future studies should prioritize the establishment of standardized experimental protocols to minimize result variability, alongside an in-depth exploration of post-translational modification mechanisms to elucidate discrepancies between transcriptional and translational levels. Furthermore, long-term clinical trials are needed to determine how exercise interventions can be tailored according to disease stages to develop personalized “precision exercise prescriptions” for patients with MASLD.

## Figures and Tables

**Table 2:** Effects of Exercise Interventions on Mitochondrial Quality Control-Related Indicators.

Reference	Study Model			Exercise Protocol	Key Results ↓ Down Significantly, ↑ Up Significantly, ↔ No Significant	
	Specie Sex	Age	Disease Stage	Exercise Classification	MASLD/MASH vs. Control	Exercise vs. MASLD/MASH
Zou et al.(2023) [23]	Zebrafish AB strain	6 months	MASLD	Aerobic Exercise MICT	MASLD: ↓ PGC1α protein ↓ TFAM protein ↓ NRF1 protein ↓ NRF2 protein ↓ PINK1 protein ↓ Parkin protein ↑ P62 protein	MICT: ↑ PGC1α protein ↑ TFAM protein ↑ NRF1 protein ↑ NRF2 protein ↔ PINK1 protein ↑ Parkin protein ↓ P62 protein
Deng et al. (2025) [17]	Rats Sprague-Dawley Male	300 g	MASLD	Aerobic Exercise HIIT or MICT	MASLD: ↓ PGC1α protein ↓ PINK1 protein ↓ Parkin protein	HIIT: ↑ PGC1α protein ↑ PINK1 protein ↑ Parkin protein MICT: ↑ PGC1α protein ↑ PINK1 protein ↑ Parkin protein
Gonçalves et al. (2016) [18]	Rats Sprague-Dawley Male	6–7 weeks	MASH	Aerobic Exercise MICT or VWR	MASH: ↔ PGC1α protein ↓ TFAM protein ↓ PINK1 protein ↓ Parkin protein ↔ LC3-II protein ↔ P62 protein	MICT: ↑ PGC1α protein ↑ TFAM protein ↑ PINK1 protein ↑ Parkin protein ↔ LC3-II protein ↔ P62 protein VWR: ↑ PGC1α protein ↔ TFAM protein ↔ PINK1 protein ↔ Parkin protein ↔ LC3-II protein ↔ P62 protein
Andani et al.(2024) [25]	Rats Wistar Male	230 ± 10 g	MASH	Aerobic Exercise HIIT or MICT	MASH: ↔ DRAM mRNA	HIIT: ↔ DRAM mRNA MICT: ↑ DRAM mRNA
Stevanović-Silva et al. (2023) [20]	Rats Sprague-Dawley Female	7 weeks	GDM MASLD	Aerobic Exercise MICT + VWR	GDM MASLD: ↔ PGC1α protein ↔ TFAM protein ↔ PINK1 protein ↓ Parkin protein ↔ LC3-II/LC3-I protein	MICT + VWR: ↔ PGC1α protein ↑ TFAM protein ↔ PINK1 protein ↔ Parkin protein ↔ LC3-II/LC3-I protein
Li et al. (2024) [26]	Mice C57BL/6J Male	12 weeks	MASLD	Aerobic Exercise HIIT or MICT	MASLD: ↓ PINK1 protein ↓ Parkin protein ↑ LC3-II/LC3-I protein ↑ BNIP3 protein	HIIT: ↔ PINK1 protein ↑ Parkin protein ↔ LC3-II/LC3-I ↓ BNIP3 protein MICT: ↑ PINK1 protein ↑ Parkin protein ↓ LC3-II/LC3-I protein ↓ BNIP3 protein
Rosa-Caldwell et al. (2017) [21]	Mice C57BL/6J Male	8 weeks	MASLD	Aerobic Exercise VWR	MASLD: ↓ PGC1α mRNA ↔ LC3-II/LC3-I protein ↔ P62 protein ↓ BNIP3 protein ↔ BNIP3 mRNA	VWR: ↔ PGC1α mRNA ↑ LC3-II/LC3-I protein ↓ P62 protein ↔ BNIP3 protein ↔ BNIP3 mRNA
da Costa Fernandes et al. (2025) [16]	Mice Swiss Male	8 weeks	MASLD	Resistance Exercise	MASLD: ↔ PGC1α mRNA ↔ TFAM mRNA ↔ NRF1 mRNA ↑ NRF2 mRNA ↑ PGC1α protein ↑ NRF2 protein	RE: ↓ PGC1α mRNA ↓ TFAM mRNA ↓ NRF1 mRNA ↓ NRF2 mRNA ↓ PGC1α protein ↓ NRF2 protein
Wang et al. (2023) [19]	Mice C57BL/6J Male	6 weeks	T2DM MASH	Aerobic Exercise HIIT	T2DM MASH: ↓ PGC1α mRNA ↓ TFAM mRNA ↓ NRF1 mRNA	HIIT: ↑ PGC1α mRNA ↑ TFAM mRNA ↔ NRF1 mRNA

In this study, the MASLD group refers to subjects with simple steatosis, distinct from the MASH group. ↑: upregulated; ↓: downregulated; ↔: no significant change. Abbreviations: BNIP3, BCL2/adenovirus E1B 19 kDa protein-interacting protein 3; DRAM, Damage-regulated autophagy modulator; GDM, Gestational diabetes mellitus; HIIT, High-intensity interval training; LC3, Microtubule-associated protein 1 light chain 3; MASH, Metabolic dysfunction-associated steatohepatitis; MASLD, Metabolic dysfunction-associated steatotic liver disease; MICT, Moderate-intensity continuous training; NRF1, Nuclear respiratory factor 1; NRF2, Nuclear respiratory factor 2; P62, Sequestosome 1; Parkin, E3 ubiquitin-protein ligase Parkin; PGC-1α, Peroxisome proliferator-activated receptor gamma coactivator 1-alpha; PINK1, PTEN-induced kinase 1; RE, Resistance training; T2DM, Type 2 diabetes mellitus; TFAM, Mitochondrial transcription factor A; VWR, Voluntary wheel running.

## Data Availability

No new data were created or analyzed in this study. Data sharing is not applicable to this article.

## Supplementary Materials

The following supporting information can be downloaded at https://www.mdpi.com/article/10.3390/metabo16010011/s1: Table S1. The specific search strings of PubMed and Web of Science; Table S2. PRISMA 2020 checklist.

## Abbreviations

The following abbreviations are used in this manuscript:

NAFLD	Non-alcoholic fatty liver disease
MAFLD	Metabolic dysfunction-associated fatty liver disease
MASLD	Metabolic dysfunction-associated steatotic liver disease
MASH	Metabolic dysfunction-associated steatohepatitis
OPA1	Optic atrophy 1
mtDNA	Mitochondrial DNA
MFN1	Mitofusin 1
MFN2	Mitofusin 2
ER	Endoplasmic reticulum
Drp1	Dynamin-related protein 1
MiD49	Mitochondrial dynamics proteins of 49 kDa
MiD51	Mitochondrial dynamics proteins of 51 kDa
Fis1	Fission protein 1
MFF	Mitochondrial fission factor
ROS	Reactive oxygen species
HIIT	High intensity interval training
MICT	Moderate intensity continuous training
VWR	Voluntary wheel running
DEX	Dexamethasone
T2DM	Type 2 diabetes mellitus
GDM	Gestational diabetes mellitus
HFD	High-fat diet
HFHS	High-fat high-sucrose diet
HIS	High-intensity speed
MCD	Methionine-choline deficient diet
MVCC	Maximal voluntary carrying capacity
RE	Resistance exercise
STZ	Streptozotocin
VO2peak	Peak oxygen uptake
OMA1	Mitochondrial inner membrane zinc-dependent metalloprotease
YME1L	Yeast mitochondrial escape 1-like ATPase
WD	Western diet
TFAM	Mitochondrial transcription factor A
PGC-1α	Peroxisome proliferator-activated receptor gamma coactivator 1-alpha
NRF1	Nuclear respiratory factor 1
NRF2	Nuclear respiratory factor 2
P62	Sequestosome 1
BNIP3	BCL2/adenovirus E1B 19 kDa protein-interacting protein 3
DRAM	Damage-regulated autophagy modulator
PINK1	PTEN-induced putative kinase 1
LC3	Microtubule-associated protein 1 light chain 3
Parkin	E3 ubiquitin-protein ligase Parkin
